# Intrathecal baclofen: Its effect on symptoms and activities of daily living in severe spasticity due to spinal cord injuries: A pilot study

**DOI:** 10.4103/0019-5413.45323

**Published:** 2009

**Authors:** Yogendrasinh Jagatsinh

**Affiliations:** Specialist Registrar, Northern Deanery, Newcastle-Upon-Tyne, UK

**Keywords:** Activities of daily living, intrathecal baclofen, spasticity, spinal cord injury

## Abstract

**Background:**

Spasticity is a major problem related to spinal cord injuries. Use of intrathecal baclofen with an implanted pump seems a very useful mode of therapy in patients in whom oral antispasmodic agents are either not effective or produce intolerable side-effects.

**Materials and Methods:**

Twenty-four patients with mean age 50 years (range 32-72 years) had intrathecal baclofen pump implanted for the severe spasticity of spinal origin. One patient died following implantation of pump due to natural causes and was not included in the study. The patients were followed up for mean 22 months (range, one to five years).

**Results:**

All 24 patients showed improvement in their spasm following the procedure. Improvement was noted in pain (12), sleep disturbance (20) and sphincter control (14). Patients had improvement in activities of daily living such as feeding ability (10), self care (10), indoor and outdoor mobility (19), and driving (4). One patient had catheter leakage immediately after the surgery and required change of catheter. The radio telemetry allows very good adjustment of the dose according the individual patients needs.

**Conclusion:**

Intrathecal baclofen pump improves the symptoms of spasm and also the quality of life. It helps the patient to live more independently. It is not an irreversible surgery for the patient and hence it is very useful in the changing the dynamics in this group of patients.

## INTRODUCTION

Spasticity is a major disorder characterized by velocity-dependent increase in the tonic stretch reflex (muscle tone) with exaggerated tendon jerks resulting from hyperexcitability of the stretch reflexes, as one component of the upper motor neuron syndrome.^1^ Troublesome spasticity is a common consequence of central nervous system injury, particularly spinal cord damage once the spinal shock stage abates.

In clinical settings, severe spasticity not only reduces rehabilitation goals of the individual but it produces ailments such as joint contractures leading to bad posture, pressure ulcers, detrusor sphincter dyssynergy and impaired motor functions. Most spinal cord injured patients with mild to moderate spasticity are effectively managed with conservative measures and available oral therapy, but the management of severe spasticity is less satisfactory. Spasticity may not always be detrimental, in that mild to moderate forms may act as a compensatory stabilizer for motor deficit, but severe spasticity is invariably detrimental as mentioned above.

It is our experience that around 10–15% of patients with spinal spasticity experience unacceptable side-effects or failure of benefits of conventional oral antispasmodic medications. Some workers have reported 25–35% rates of such patients.^2^

Fortunately, the abundance of GABA-B receptors in the superficial layers of the spinal cord^3^ means that intrathecal administration of baclofen results in a greater than four-fold increase in it's cerebra spinal fluid (CSF) level with 1% of the oral dose. In CSF, baclofen has a half-life of five hours and a duration of action of ten to twelve hours with very little drug returning to the systemic circulation.^4^ Measurement of CSF baclofen concentration shows lumbar to cervical ratio of 4:1, thus avoiding untoward cerebral effects of the intrathecal administered baclofen.

An implanted programmable drug pump is essential to deliver this therapy. It allows continuous administration of the drug, precise adjustment of the dose, and variation of the dosage schedules such as the circulation rhythm used to reduce night-time spasms.

## MATERIALS AND METHODS

**Figure 1:**
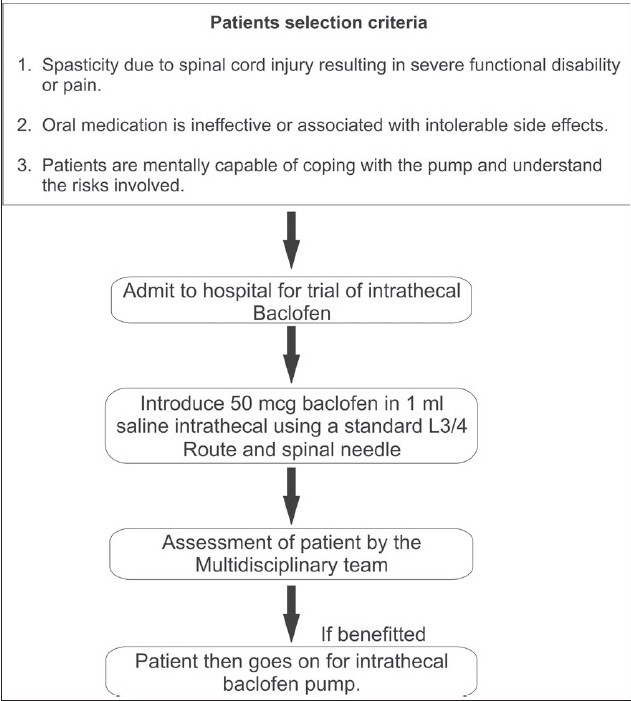
A line diagram depicting the protocol for assessment of suitability of intrathecal baclofen

From January 2002 to December 2006, thirty-seven adult subjects with diagnoses of severe spasticity due to spinal cord origin had intrathecal baclofen (ITB) pumps implanted (Synchromed I and II, Medtronic Inc, USA).^5^ The patients who had persistent uncontrolled spasticity despite oral antispasmodic medication were offered implantation of ITB pumps. The protocol for assessment of suitability of ITB is highlighted in Figure 1. Exclusion criteria included patients with other co-morbidities and patients who refused to give consent. The aim of the study was to find out whether implantation of the pump had actual benefits in these patients in relieving symptoms and functional improvement in their activities of daily living. Further, any beneficial effect of this therapy on bladder and bowel function was also noted.

Prior to surgery, the most comfortable body for area for the implantation of the ITB pump is selected which is usually under the skin below the belt-line to one side of the lower abdomen.

There are two choices of Synchromed II pump differing in the size of the drug reservoir, i.e. either 20 or 40 mL. The choice of pump depends on many factors including the size of the patient, the anticipated daily dose of ITB, and the estimated refill intervals.

The catheter is tunneled through a Touhey's needle inserted through L3/4 space by making a small incision on the back. The catheter is guided upwards to the level of sixth thoracic vertebra and is checked on fluoroscopy. After this, the catheter is then tunneled beneath the skin around the flank of the patient to the lower abdomen. The pump is then implanted just beneath the skin in the subcutaneous fatty tissue, which lies above the abdominal muscles of the lower abdomen. The pump is connected to a small flexible thin catheter, which is tunneled beneath the skin around the flank of the patient and into the intrathecal space containing the cerebrospinal fluid. The pump is then filled with baclofen and the dose is adjusted with radiotelemetry.

This was a single-site open-label pilot study. The questionnaire was designed by the author using various outcome measure tools such as Barthel index,^6^ Functional Independence measure,^7^ Spinal cord Independence measure.^8^ The questionnaire was sent to 37 patients, out of whom 24 patients responded and one patient had died after the procedure due to natural causes. The data was entered into the Access Database and analysed using Excel by the author. Demographic variables were compared using the Fisher exact test and *t* test.

## RESULTS

There were 17 males and 7 females in the study. The age range was 32–72 years with a mean of 50 years. All 24 patients showed improvement in the Penn's Spasm score^9^ following the pump. Pre implantation, 14 patients had their sphincters affected due to spasm in the form of bypassing and leaking. Post implantation, 9 patients reported improved bladder/bowel function and the rest of them had no change. We do not exactly know the reason for improvement in visceral function but we assume it is due to relaxation of bladder and bowel smooth muscles and relief of detrusor sphincter dyssynergia. The improvement is noted immediately after ITB, but in addition to achieving functional abilities, bladder and bowel function also improved in 9 patients [Table 1]. The ITB helped in many situations such as bypassing and expelling the catheters out due to spasm. It was possibly a byproduct of relaxation of the sphincters and detrusor muscles. We have used the (VAS) Visual Analog Scale and improvement in scale of 2 was considered significant. [Table 2].

**Table 1:** Effect of intrathecal baclofen on activities of daily living

Activities of daily living	Before ITB	After ITB
Feeding ability	10 (Rest of them were not able to feed themselves due to Tetraplegia)	8 Improved
		2 No change
Hygiene and self-care	10	9 Improved
		1 No change
Dressing/undressing	16	14 Improved to self-dressing
		2 No change
Bladder/bowel function	14	9 Improved
		5 No change
Transfers	16	15 Improved
		1 No change
Mobility (indoors/outdoors)	19	16 Improved
		2 No change
		1 Worse
Social/recreational activities	17	13 Improved
		3 No change
		1 Worse
Driving ability	4	3 Improved
		1 Worse

**Table 2:** Effect of intrathecal baclofen on patients symptoms

Symptoms	Before ITB* (No. of patients)	After ITB* (No. of patients)
Pain	12	10 Improved (Improvement of 2 on VAS)
		2 No change
Strength	16	9 Improved
		6 No change
		1 Worse
Co-ordination/Dexterity	18	17 Improved
		1 Worse
Sleep	20	18 Improved
		1 No change
		1 Worse

* ITB: intrathecal baclofen

The one patient who showed worsening of the symptoms had catheter leakage and underwent further investigations and catheter change. He showed improvement following the re-citation of the catheter.

## DISCUSSION

In neurological terms, spasticity is the result of a decreased threshold of responsiveness of motor neurons to proprioceptive input. There are several possible sites of action within the stretch reflex which can cause exaggerated stretch activity and produce muscle hypertonia in spastic conditions. These include (1) muscle spindle hyperactivity due to increased gamma motor neuron activity; (2) decreased pre-synaptic inhibition due to impaired descending influence on segmental input, or structural reorganization of the propriospinal input (sprouting in primary sensory neuron terminals); (3) alteration in segmental reflex mechanisms with resulting increased responsiveness of alpha and gamma motor neurons to the input from muscle afferents; (4) impaired suprasegmental influence on alpha motor cells and consequent hyperactivity of alpha motor cells.^10^ In the case of a sustained (chronic), functionally complete injury of the spinal cord in which the spinal cord is divided from the neural influence of the brain, the characteristic neurological findings are muscle hypertonia predominantly in the flexor muscle group, moderately enhanced tendon jerks, and sometimes short lasting clonus.^11^

Any kind of additional continuous afferent inflow to the spinal cord, as is the case in patients with pressure sores, urinary tract dysfunctions, constipation, etc. temporarily increases the excitability of the spinal cord and maintains the tendon reflex responsiveness, even when these reflexes are elicited repeatedly with constant strength stimuli.

Intrathecal use of baclofen for severe spasticity was reported by Penn and Kroin in 1984.^12^ Since, then several studies supporting this have been published by various authors.^13^–^17^ Intrathecal baclofen has been found to improve supraspinal spasticity, although our experience in this aspect is limited.^18^ Similar to previously published studies, we have observed improvement in the bladder functions in spinal cord injured patients following ITB implantation.^19^–^22^

The site of action of baclofen in the spinal cord is not clear. baclofen, first used in humans in 1974,^23^ is a gamma-amino butyric acid (GABA) analogue that binds to the GABA-B receptor, resulting in the inhibition of calcium influx into presynaptic terminals and thus suppressing the release of excitatory neurotransmitters. These GABA receptor sites occur widely throughout the central nervous system.^24^–^26^

These findings are consistent with observations of profound suppression of both mono and polysynaptic reflexes with ITB in animal models,^27^–^30^ although it is argued that intrathecal baclofen effectively suppresses segmental monosynaptic reflexes, such as the tendon jerk, muscle stretch reflex and the H-reflex.

Literature reviewed so far suggests satisfactory outcomes using intrathecal baclofen in the management of severe spasticity. The implantable drug delivery systems^5^ have performed satisfactorily in this severely disabling and persistent spasticity. It is a reversible therapy and with radio telemetry dosage adjustment, it makes this therapy very versatile and non-invasive. There is a need to develop smaller sized pumps for management of severe spasticity in children with spinal cord injury. The Medtronic programmable pump seems to have performed far better compared to mechanical and constant infusion pumps.

As complications are related to operator inexperience, it is recommended that specific center or personnel's should only perform this procedure where protocols can be standardized and long-term results properly evaluated. All the patients treated in this centre have shown benefits as a result of the intrathecal baclofen and now have improved quality of life as a result of this therapy. In our center, this has replaced orthopedic and neurosurgical intervention for these cases suffering with irreversible severe spasticity.

Continued research is needed in the management of spasticity in spinal cord injured patients. With the advent of therapy to treat severe spasticity, not only will it help these patients to achieve further independence in their activities of daily living, but it will also relieve discomfort and medical problems resulting as a consequence of severe spasticity. Such research could be in the field of neural regeneration, neural implantation or in the field of local or distal electrical stimulation.
